# Factors Associated with Dietary Habit Changes in Korean Stomach Cancer Survivors after Cancer Treatment

**DOI:** 10.3390/nu15143268

**Published:** 2023-07-24

**Authors:** Junhee Park, Jiyoung Kim, Dong Wook Shin, Jinyoung Shin, Belong Cho, Yun-Mi Song

**Affiliations:** 1 Department of Family Medicine & Supportive Care Center, Samsung Medical Center, Sungkyunkwan University School of Medicine, Seoul 06351, Republic of Korea; junhee.park26@gmail.com (J.P.); dwshin.md@gmail.com (D.W.S.); 2 Department of Family Medicine & Health Promotion Center, Seoul National University Hospital, Seoul 03080, Republic of Korea; a7071@snuh.org (J.K.);; 3 Department of Clinical Research Design and Evaluation, Samsung Advanced Institute of Health Science and Technology, Sungkyunkwan University, Seoul 06355, Republic of Korea; 4 Department of Family Medicine, Konkuk University Medical Center, Seoul 05030, Republic of Korea; jyshin@kuh.ac.kr

**Keywords:** stomach cancer survivor, dietary habit change, nutritional guidelines

## Abstract

The current nutritional guidelines for stomach cancer survivors (SCSs) mainly focus on the influence of the surgical resection of the stomach, with limited guidance regarding a wider range of food options. We aimed to investigate the factors associated with healthier dietary changes in Korean adult SCSs. This cross-sectional study assessed dietary pattern changes after cancer treatment for 11 food categories, using a self-administered questionnaire. A ‘healthier dietary change’ was operationally defined as a reduced consumption of red and processed meat, grains, salt, and burnt food, and an increased consumption of poultry, fish, vegetables, fruits, legumes, and dairy products. Among a total of 624 SCSs, approximately 60% of participants reported dietary changes in a healthier direction in three or more food categories, while 9.1% reported no changes. There was no significant difference in dietary habit changes between surgery types. Multivariable adjusted analysis showed that elderly and long-term survivors were inversely associated with a healthier dietary change. SCSs with a higher level of educational achievement and income were more likely to make healthier changes in their intake of processed meat, vegetables, fruits, burnt food, or salt. SCSs with higher levels of fear of cancer recurrence, anxiety, or depression were more likely to follow healthier dietary changes regarding fish, meat, fruits, grains, or burnt food. Change in dietary pattern varied across different food items, and was associated with various characteristics of SCSs. It is crucial to repeatedly provide SCSs with information about healthier dietary patterns, considering their sociodemographic, clinical, and psychological characteristics.

## 1. Introduction

Stomach cancer is globally ranked as the fifth most prevalent cancer in terms of incidence, and the fourth in terms of cancer mortality [1]. The East Asian population, in particular, exhibits a higher incidence of stomach cancer [1]. In Korea, 25,768 new cases of stomach cancer were registered in 2021 [2]. Notably, the 5-year survival rate of stomach cancer patients has remarkably improved from 43.9% in 1993–1995, to 77.5% in 2015–2019 [3]. This improvement can be attributed to advancements in the early diagnosis and treatment strategy, leading to an increasing number of stomach cancer survivors (SCSs). SCSs often face challenges related to poor dietary patterns or inadequate nutrient intake, primarily resulting from the surgical resection of the stomach. Commonly reported health problems [4,5] of SCSs include weight loss, dyspepsia, gastrointestinal reflux disease, dumping syndrome, anemia, and osteoporosis [6,7]. A malabsorption or deficiency of micronutrients such as vitamins, calcium, and iron could be related to these health issues [8]. Consequently, SCSs have unique dietary needs [4] when it comes to evidence-based guidelines specifically tailored to them, addressing the metabolic effects and biochemical interactions related to the malabsorption of nutrients [9]. Such guidelines are crucial for maintaining an optimal nutritional status, preventing the development of new diseases, reducing cancer-specific and overall mortality, and enhancing the quality of life of SCSs.

Despite the growing body of evidence highlighting the need for dietary support among cancer survivors [10], it seems unclear who to target, and how to effectively recommend modifications in dietary patterns. The current nutritional management of patients with stomach cancer mainly revolves around the extent of the surgical resection of the stomach, with the involvement of the esophageal or pyloric sphincter [11]. SCSs are often advised to eat smaller, more frequent meals or snacks, rather than eating three times a day, and to avoid drinking liquids with meals. Eating a diet high in protein and fiber, and low in simple carbohydrates and sugars, is also recommended, to help manage the symptoms of dumping syndrome and dyspepsia [12]. However, it is widely recognized that cancer survivors are likely to encounter multiple barriers that impact their adherence to nutritional recommendations, such as clinical (i.e., chronic pain, altered sense of food taste, indigestion [13], and metabolic changes in the gastrointestinal hormones of energy homeostasis [14,15]) or environmental (i.e., a low cognition of risk behaviors [16], and family/social support [17]) barriers. Therefore, helping survivors make sustainable behavior changes to follow the guideline, with consideration given to the barrier factors, is also important. However, there is a lack of evidence regarding which factors are associated with dietary behavioral change among SCSs. Besides, given the difference in dietary patterns between Western and Asian people, the development of specific guidelines for the desirable dietary intake of Asian cancer survivors is necessary [18].

In this regard, we conducted this study to investigate how Korean SCSs change their dietary intake pattern after the completion of cancer treatment. We also evaluated the factors associated with healthier dietary intake changes in Korean SCSs.

## 2. Materials and Methods

### 2.1. Study Participants

The participants of this cross-sectional study were a total of 624 Korean adult (≥19 years) SCSs who had completed primary cancer treatment. We recruited study participants at two university-affiliated hospitals between September 2014 and February 2017. Participants were recruited irrespective of the time that had elapsed since the initial cancer diagnosis, when they visited the hospital for routine surveillance after cancer treatment, or to seek consultation for health issues. These two hospitals are reported to provide medical services to around 20% of Korean cancer patients. Among the initially enrolled 689 SCSs, we excluded 65 for the following reasons: those who had multiple cancers (n = 40); who had recurrent or metastatic cancer (n = 7); and who did not receive surgery (n = 2) or any treatment (n = 8); or who did not respond to the questions asked about dietary habits (n = 8).

### 2.2. Dietary Intake Pattern

We assessed the dietary pattern changes in the study participants using a self-administered questionnaire with five response levels (decreased a lot, decreased somewhat, did not change, increased somewhat, increased a lot). We asked if, after cancer treatment, there were any changes in their total amount of food intake, as well as if there were any changes in the frequency of intake for 11 specific food items (provided as a Supplementary Table S1). The 11 individual food categories included red meat (pork and beef), poultry (chicken and duck), processed meat (sausage, ham, and bacon), fish, vegetables, fruit, beans, dairy products, grains, salt, and burned food. We did not survey refined grains separately from whole grains. Additionally, the participants were asked if there had been any changes in their overall meal size after cancer treatment.

In accordance with the cancer prevention recommendations of the World Cancer Research Fund (WCRF)/American Institute for Cancer Research (AICR) [19], we established a “healthier dietary change” as follows: (1) a reduced consumption of red and processed meat, grains, salt, and burnt food; (2) an increased consumption of poultry, fish, vegetables, fruit, legumes, and dairy products as valuable sources of protein and micronutrients. We viewed a decrease in grain intake as a healthier dietary change, given that Asians tend to consume extremely high-carbohydrate daily diets (>70% of energy), consisting of various refined grains such as white rice and noodles [20,21], compared with the Western population [22]. In addition, we calculated a dietary change score for each participant, by tallying the total number of dietary changes in a healthier direction across 11 individual food categories.

### 2.3. Other Study Variables

The cancer-related clinical information was obtained through a review of medical records, which included the age at cancer diagnosis, time since cancer diagnosis (<1 year, 1–4 years, 5–9 years, and ≥10 years), cancer stage (I, II, III, or IV), and cancer treatments received (surgery, chemotherapy, or radiotherapy). The surgical procedures encompassed various stomach operations, such as a total gastrectomy, subtotal gastrectomy, wedge resection, and endoscopic submucosal dissection. We collected data on the preoperative weight and height by reviewing medical records. The weight and height at the time of survey were measured to the nearest 0.1 kg and 0.1 cm, respectively. The measurements were taken using a standardized scale and stadiometer, while participants were wearing light clothing and no shoes. Subsequently, we calculated the body mass index (BMI) as weight divided by height squared (kg/m^2^), which was categorized into four groups (<18.5 kg/m^2^, 18.5–22.9 kg/m^2^, 23–24.9 kg/m^2^, and ≥25 kg/m^2^) [23].

We collected information on sociodemographic and psychological factors using a self-administered questionnaire. Marital status was assessed by determining whether the participant was living with their spouse/partner or not. The household monthly income was categorized into three groups (KRW ≥ 4,000,000, KRW 2,000,000–3,999,999, or KRW < 2,000,000). Educational achievement was categorized into three groups (≤middle school, high school, ≥college), considering the Korean education system. Smoking status was classified into three groups: never, ex-smoker, and current smoker. Alcohol consumption was classified into two groups: currently and not currently drinking.

To evaluate the participant’s psychological status, we utilized the Korean version of the Hospital Anxiety and Depression Scale (HADS) to assess participants’ psychological status. The HADS is a validated self-rating report assessment, comprising fourteen items designed to measure anxiety (HADS-A) and depression (HADS-D) [24]. We adopted HADS-A and HADS-D ≥ 8 as a cut-off value for detecting anxiety and depression [24]. The severity subscale of the fear of cancer recurrence index (K-FCRI), known as the short form of FCRI, was employed to assess the fear of cancer recurrence. Cronbach’s alpha coefficient for the FCRI short-form Korean version was 0.77 [25], indicating a good internal consistency. A score ≥ 13 on the severity subscale (short-form FCRI) was used as a cut-off score to determine clinically significant levels, following the recommendation by Simard et al. [26].

### 2.4. Statistical Analysis

We investigated whether changes in dietary patterns after cancer diagnosis varied based on the age at cancer diagnosis, and the time that had elapsed since cancer diagnosis, using the chi-squared test. Furthermore, we analyzed the association between healthier dietary pattern changes after cancer diagnosis with various sociodemographic, clinical, and psychological factors, by estimating the odds ratio (OR) and 95% confidence intervals (CI) from multiple logistic regression analysis. SPSS version 24 (IBM Corp., Armonk, NY, USA) and SAS software 9.4 (SAS Institute Inc., Cary, NC, USA) was used for all statistical analyses. A two-sided *p*-value of less than 0.05 (<5%) was considered statistically significant for all analyses.

## 3. Results

### 3.1. Baseline Characteristics

Out of the total 624 participants, 58% were male, and the mean age at the time of the survey was 59.2 ± 9.9 years old. The mean age at cancer diagnosis was 52.5 ± 10.2 years old, and the mean duration since cancer diagnosis was 6.7 ± 3.0 years. Stage 1 stomach cancer accounted for 67.8% of the cases. All participants underwent surgical treatment, with 28.7% also receiving chemotherapy, and 12.2% receiving radiotherapy in addition. Biloth-1 subtotal gastrectomy was the most common type of surgery, followed by total gastrectomy (22.8%), and Biloth-2 subtotal gastrectomy (Table 1).

### 3.2. Distribution of Dietary Habit Change

**Figure 1:**
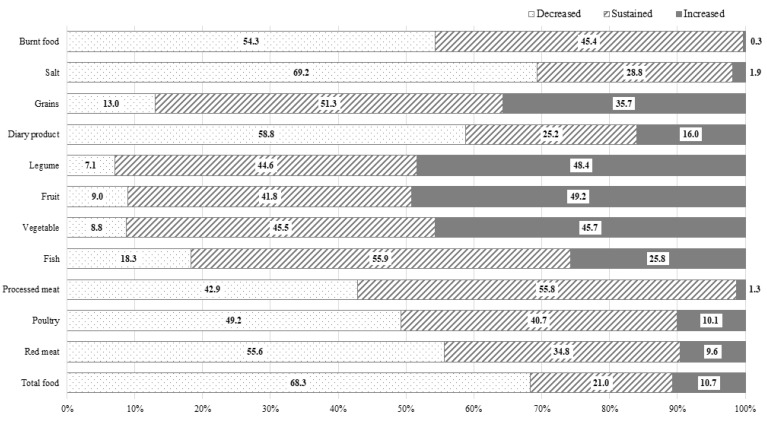
Dietary habit changes after cancer diagnosis in 624 Korean stomach cancer survivors.

Approximately two-thirds (68.3%) of participants reported a reduction in their overall food consumption. Around half of the participants indicated a decrease in their intake of red meat, processed meat, poultry, dairy products, salt, and burnt food. Conversely, around half of the participants also reported an increased intake of vegetables, fruit, and legumes. The most common food category for which healthier dietary change was reported was salt, followed by red meat, burnt food, fruit, legumes, vegetables, and processed meat (Figure 1). Around 60% experienced a shift toward a heathier dietary pattern in three or more categories of food, while 9.1% did not report any positive dietary changes. With the increase in age at cancer diagnosis, the number of food items that changed into a healthier intake pattern tended to decrease (P trend < 0.05) (Supplementary Figure S1). A reduction in total food intake was most common in SCSs who had undergone a pylorus-preserving gastrectomy (79.4%), followed by total gastrectomy (73.9%), Biloth-2 subtotal gastrectomy (70.7%), and Biloth-1 subtotal gastrectomy (64.6%). There was no significant difference in dietary habit changes between surgery types (Supplementary Table S2).

### 3.3. Dietary Habit Changes Stratified by Age and Time Elapsed after Cancer Diagnosis

As the age at cancer diagnosis increased, a higher proportion of participants, especially female SCSs, reduced their vegetable and fruit intake. On the other hand, fewer participants, regardless of sex, reduced their intake of processed meat and burnt food as their age at cancer diagnosis increased. In males, the proportion of SCSs who reduced their salt intake decreased with an increase in the age at cancer diagnosis. (Supplementary Table S3).

As the time that had elapsed after cancer diagnosis increased, more participants, particularly female SCSs, maintained or increased their intake of vegetables, fruits, and legumes, compared to the preoperative period. Conversely, as the time elapsed after the cancer diagnosis increased, fewer participants reduced their intake of salt and grains, and this trend was evident only in male SCSs. However, there were no significant changes in the intake of red meat, processed meat, poultry, fish, and burnt food with the increase in the time elapsed after cancer diagnosis (Supplementary Table S4).

### 3.4. Factors Associated with Dietary Habit Changes in a Healthier Direction

Table 2 presents the multivariable-adjusted association between changes in dietary patterns in a healthier direction after cancer diagnosis, and various sociodemographic, clinical, and psychological factors. Each 1-year increase in age was inversely associated with a healthier dietary change in the consumption of poultry, processed meat, vegetables, fruit, legumes, and burnt food. Female SCSs exhibited a decreased likelihood of reducing their burnt food intake, compared to male SCSs. SCSs who were living with a spouse were less inclined to reduce their grain intake compared to those without a spouse. SCSs with a higher level of educational achievement were more likely to change their dietary habits in a healthier direction in terms of processed meat, vegetables, fruit, and burnt food, compared to those with a lower education level. Participants with a higher income tended to change their dietary habits in a healthier direction regarding the intake of vegetables, fruit, and salt.

As the time elapsed after the cancer diagnosis increased, SCSs were less likely to increase their intake of fish, and less likely to reduce their intake of grains. SCSs who underwent a Biloth-1 subtotal gastrectomy were more likely to increase their vegetable intake compared to those who had a total gastrectomy. SCSs who received chemotherapy were more likely to increase their poultry consumption, whereas those who received radiotherapy were less likely to increase their poultry consumption. Participants who were overweight or obese at cancer diagnosis showed a higher probability of reducing their intake of red meat, processed meat, and grains than participants who were a normal weight or underweight.

Furthermore, participants with a higher FCR were more likely to increase their intake of fish and fruit compared to those with a lower FCR. Participants with anxiety had a greater likelihood of reducing their grain consumption, while those with depression were more likely to reduce their red meat, processed meat, and burnt food consumption.

## 4. Discussion

To the best of our knowledge, the present study is the first to investigate the dietary pattern changes among SCSs after cancer treatment and its associated factors. Our findings revealed that nearly all the SCSs made dietary pattern changes in a healthier direction for at least one type of food, with over half of them modifying their diet for multiple food categories. Most SCSs reported a reduction in their total food consumption after their cancer diagnosis, which is consistent with previous finding [27]. A study of Australian survivors of breast, colorectal, and hematological cancers found that 55% of the study participants made dietary changes after their cancer diagnosis [28]. In the Australian study, the most common changes included increasing fruit and vegetable intake (36%), followed by reducing intake of red meat (25%), sugar (20%), fat (12%), and dairy products (7%) [28]. The direction of dietary pattern change in our study appears to be similar. However, the proportion of survivors who made dietary changes was notably higher in our study. We believe this difference could be attributed to the different distribution of cancer types between the two studies, considering the stomach’s significance as a major digestive organ. In fact, SCSs exhibited a greater emphasis on calorie intake from potatoes, starches, legumes, seeds, vegetables, and fruit, compared to other cancer survivors in a previous Korean study [18].

The Continuous Update Project (CUP) conducted by WCRF/AICR argued that any effect of salt on stomach cancer principally came from the regular consumption of salt and salt-preserved foods [29]. The high concentration of sodium chloride in these foods can cause mucosal damage, leading to a heightened susceptibility to mutagenesis or cancer development [30]. Additionally, a high salt intake may stimulate the colonization of *Helicobacter pylori*, which is known as the strongest risk factor for stomach cancer [31]. In our study, around 70% of SCSs reported a reduction in their salt intake, ranking it as the most commonly changed dietary item (Figure 1). However, another 30% did not decrease their salt intake, and 1.9% even reported an increase in their salt intake after their cancer diagnosis. Notably, a higher income was strongly associated with a reduced salt intake. We presume that this inverse association between income and salt intake may reflect socioeconomic disparities in access to fresh food [32]. Interestingly, with a longer time lapse after cancer diagnosis, SCSs were significantly less likely to reduce their salt intake. This finding indicated that as the duration of survival increased, cancer survivors became less keen on healthcare maintenance. A similar phenomenon has also been observed regarding smoking behaviors [33]. Therefore, it is crucial to consistently provide health education around desirable health behavior, regardless of the time elapsed since a cancer diagnosis.

The WCRF recommends that cancer patients limit their red meat consumption to no more than about 350 to 500 g, and consume very little, if any, processed meat, while encouraging the intake of poultry and fish as a valuable substitute [34]. The consumption of red and processed meat has been suggested to increase the risk of non-cardia stomach cancer [35], primarily due to the presence of sodium nitrate and nitrite used as preservatives added into products such as ham, bacon, and sausage. The compounds can react with amino acids to produce carcinogens, such as N-nitro compounds, in the stomach [36]. In our study, only 42.9% and 55.6% of SCSs reported reducing their intake of processed meat and red meat, respectively, and preoperatively overweight or obese patients were more likely to decrease such consumption. In addition, 49.2% of SCSs reduced their poultry intake. While the influence of poultry and fish on cancer risk is still inconclusive [29], given the role of processed meat and red meat as significant risk factors for colon cancer and atherosclerotic diseases, it is advisable to strongly recommend reducing consumption of processed meat and red meat for SCSs, and promote an increased consumption of poultry and fish as appropriate protein sources.

There is only limited evidence suggesting that consuming little or no fruit may increase the risk of stomach cancer [37]. Citrus fruits have been proposed to potentially decrease the risk of stomach cardia cancer, perhaps because of their vitamin C content [38]. The effects of vegetables including dietary fiber, and legumes on stomach cancer risk are inconclusive [29]. Furthermore, research on the relationship between stomach cancer and the beneficial effect of dietary flavonoids [39], such as polyphenol [40], isoflavones [41], and anthocyanin [42] has been continuously reported. Despite the lack of definite evidence, support for the beneficial role of vegetables, fruit, and legumes for overall health for SCSs could still be encouraged. In our study, approximately 45% of SCSs reported an increased vegetable, fruit, and legume intake, while the remaining 40% maintained their previous intake. Higher levels of education and income, and higher fear of cancer recurrence were positively associated with these dietary changes.

The relationship between dairy products and stomach cancer has been evaluated, with inconsistent findings. According to a meta-analysis, dairy intake has been inversely associated with gastric cancer in Europe and the United States, but not in Asia [43,44]. Meanwhile, there is strong evidence that the consumption of dairy products is associated with a decreased risk of colorectal cancer, but an elevated risk of prostate cancer [19]. Since SCSs may have an increased risk for colon cancer as a secondary cancer [45], an increased dairy product consumption may be regarded as a healthier diet in SCSs. Notwithstanding this, approximately 60% of SCSs in our study reported having reduced their dairy product intake, while only 16% increased their consumption. This finding contrasts with an Australian study where only 7% of participants specifically avoided dairy products [28]. Therefore, it may be necessary to further encourage Korean SCSs to increase their dairy product intake.

Older adults generally tend to consume less fast food, and more fruit and vegetables, compared to younger adults [46]. However, our study found that older SCSs at the time of cancer diagnosis were less likely to follow healthier directional dietary changes, compared to younger SCSs. As the age at cancer diagnosis increased, there was a decreasing likelihood of adopting healthier dietary habits by increasing the intake of poultry, vegetables, fruit, and legumes, while there was increasing likelihood of consuming more processed meat and burnt food, even after adjusting for education levels. This may be attributed to gastric resection having a worse influence on older SCSs than younger SCSs, leading to more frequent gastrointestinal problems, such as indigestion, and a decreased appetite with aging [47]. Thus, it is possible that the higher prevalence of gastrointestinal problems in older SCSs explains their lower intake of vegetables and fruit, compared to younger SCSs. Therefore, healthcare professionals caring for older SCSs should pay close attention to gastrointestinal symptoms and signs of malnutrition.

Interestingly, our finding that more of the subjects with higher education changed their dietary habits in a healthier direction was consistent with the findings of other studies [13,48]. It is well-established that individuals with a higher socioeconomic status tend to adhere to health recommendations better, likely due to the higher chance of health education and greater accessibility to healthcare [49,50]. Therefore, it may be necessary to make a special effort to provide health information on a healthier dietary intake to cancer survivors who may have had limited chances of health education.

With increasing survival time, the SCSs in our study were more likely to increase their grain and salt intake. Similarly, patients who have undergone sleeve gastrectomy for weight reduction have reported that initial difficulties in consuming certain foods, such as red meat, rice, and pasta, improved over time, particularly after 5 years [12,51]. Therefore, health professionals should emphasize the importance of maintaining a healthy diet for long-term cancer survivors.

Nutritional management for individuals who have had stomach cancer depends on which part of the stomach was surgically removed or altered [52]. If the pyloric sphincter is affected, it can lead to symptoms such as reflux and rapid food transit through the stomach. Thus, we assumed that the type of surgery would be associated with dietary changes. However, apart from the vegetable intake, we did not find significant differences in the dietary pattern changes among the various surgical types. This may be partly explained by post-surgical complications, such as gastrointestinal reflux disease. There also might be a difference in the change in the size of one portion, or the frequency of eating, according to surgery type, which we did not investigate in this study.

A balanced and healthy diet has been shown to be beneficial in relieving cancer survivors’ FCR [53]. In our study, SCSs with a higher fear of cancer recurrence, anxiety, and depression were more likely to change their dietary intake in a healthier direction. This finding is compatible with a Duchy study of colorectal cancer survivors that found that those who experienced symptoms of anxiety or depression were more likely to express a need for dietary support than those who without such symptoms (27.6% and 28.7%, respectively) [54]. These findings suggest that the psychological difficulties of cancer survivors, including fear, anxiety, and depression, may serve as motivators for pursuing a healthier dietary intake. However, it is crucial to implement strategies that further encourage a healthier dietary intake among SCSs who suffer from psychological difficulty.

There were several limitations that should be acknowledged in our study. Firstly, we were unable to include a non-cancer group for comparison, which limited our ability to assess differences in dietary habits between cancer survivors and people without cancer. Secondly, the study was based on a self-reported survey, so there could be recall bias. In addition, we could not evaluate the validity of the questionnaire used to assess dietary pattern changes in the present study. Thirdly, the evaluation of dietary habit changes was based on tendency, rather than precise quantitative measures. We could not assess altered nutrient absorption, nutritional status, and the association of dietary pattern change and biological change, due to the lack of data. Fourthly, we were unable to investigate the underlying reasons behind the observed associations. Finally, it is a cross-sectional study that hinders accurate causal association. Despite this limitation, our study was the first to attempt to elucidate the factors associated with healthy dietary changes in SCSs (Supplementary Table S5). In addition, we also took into consideration a comprehensive range of variables, including socioeconomic and psychological factors, in addition to cancer-related factors.

## 5. Conclusions

The present study confirmed that the majority of SCSs had a strong desire to adapt their dietary pattern in a healthier direction after cancer diagnosis. However, as time passes since cancer diagnosis, the SCSs became less inclined to maintain healthier dietary patterns for certain food items. The changes in dietary patterns differed across specific food items, and were associated with various characteristics in the SCSs. The factors identified in our study, such as a higher age, longer time lapse after cancer diagnosis, low socioeconomic status, and psychological characteristics, should be taken into account to identify stomach cancer survivors who may need more dietary guidance.

Making this effort may further assist in the provision of personally tailored dietary recommendation for stomach cancer survivors, in addition to the assessment of biomarkers and nutritional status.

## Figures and Tables

**Table 1:** Sociodemographic and clinical characteristics of the study participants: 624 Korean stomach cancer survivors.

Sociodemographic Characteristics	
Age	59.2 ± 9.9
<55 years	190 (30.4)
55–59 years	129 (20.7)
60–64 years	140 (22.4)
≥65 years	165 (26.4)
Male	362 (58.0)
Live with spouse/partner	530 (84.9)
Monthly household income	
KRW < 2 million	89 (14.3)
KRW 2–3 million	134 (21.5)
KRW ≥ 4 million	217 (34.8)
Unknown	184 (29.5)
Education achievement	
≤Middle school	70 (11.2)
High school	211 (33.8)
≥College	189 (30.3)
Unknown	154 (24.7)
Smoking status	
Never smoked	352 (56.4)
Ex-smoker	230 (36.9)
Current smoker	30 (4.8)
Unknown	12 (1.9)
Alcohol consumption	
Not currently drinking	442 (70.8)
Currently drinking	182 (29.2)
Clinical characteristics	
Age at cancer diagnosis	52.5 ± 10.2
<45 years	138 (22.1)
45–54 years	231 (37.0)
≥55 years	255 (40.9)
Time elapsed after diagnosis	6.7 ± 3.0
<1 years	14 (2.2)
1–4 years	152 (24.4)
5–9 years	395 (63.3)
≥10 years	63 (10.1)
Stage of cancer	
Stage 0	7 (1.1)
Stage 1	423 (67.8)
Stage 2	102 (16.3)
Stage 3	60 (9.6)
Stage 4	9 (1.4)
Unknown	23 (3.7)
Type of surgery received	
Total gastrectomy	142 (22.8)
Subtotal gastrectomy	470 (75.3)
Biloth-1 subtotal gastrectomy	328 (52.8)
Biloth-2 subtotal gastrectomy	75 (12.0)
Pylorus-preserving surgery	63 (10.1)
Not specifically stated	4 (0.6)
Wedge resection	6 (1.0)
Endoscopic submucosal dissection	3 (0.5)
Unknown	3 (0.5)
Type of cancer treatment received	
Chemotherapy	179 (28.7)
Radiotherapy	76 (12.2)
Preoperative body mass index	23.8 ± 3.1
<18.5 kg/m^2^	13 (2.1)
18.5–22.9 kg/m^2^	252 (40.4)
23–24.9 kg/m^2^	140 (22.4)
≥25 kg/m^2^	198 (31.7)
Unknown	21 (3.4)
Psychological characteristics	
High fear of cancer recurrence (FCRI ≥ 13)	206 (33.0)
Depression (HADS-D ≥ 8)	270 (43.3)
Anxiety (HADS-A ≥ 8)	91 (14.6)

FCRI, Fear of Cancer Recurrence Inventory. Data are presented as number (%) or mean ± standard deviation.

**Table 2:** Factors associated with dietary habit changes in a healthier direction: 624 Korean stomach cancer survivors.

Variable	Decreased Red Meat	Increased Poultry	Decreased Processed Meat	Increased Fish	Increased Vegetables	Increased Fruit	Increased Legumes	Increased Dairy Products	Decreased Grains	Decreased Salt	Decreased Burned Food
Sociodemographic characteristics											
Age at the time of cancer diagnosis											
<45 years	1.00	1.00	1.00	1.00	1.00	1.00	1.00	1.00	1.00	1.00	1.00
45–54 years	1.37 (0.85, 2.19)	0.77 (0.38, 1.54)	0.68 (0.41, 1.11)	1.77 (1.05, 2.98)	0.68 (0.41, 1.11)	0.87 (0.53, 1.44)	0.69 (0.41, 1.13)	1.44 (0.78, 2.66)	0.78 (0.38, 1.59)	1.16 (0.68, 1.98)	0.96 (0.57, 1.62)
≥55 years	1.56 (0.92, 2.63)	0.43 (0.19, 1.01)	0.41 (0.24, 0.71)	0.82 (0.45, 1.50)	0.43 (0.24, 0.74)	0.46 (0.26, 0.80)	0.47 (0.27, 0.82)	1.23 (0.61, 2.48)	0.90 (0.42, 1.95)	0.94 (0.53, 1.67)	0.54 (0.31, 0.96)
Continuous increase by 1 year	1.01 (0.99, 1.04)	0.96 (0.93, 0.99)	0.97 (0.95, 0.99)	0.99 (0.96, 1.01)	0.97 (0.94, 0.99)	0.96 (0.94, 0.98)	0.96 (0.94, 0.98)	0.99 (0.97, 1.02)	0.99 (0.96, 1.02)	0.99 (0.97, 1.01)	0.97 (0.95, 0.99)
Female (vs. male)	1.01 (0.61, 1.66)	0.94 (0.41, 2.12)	1.01 (0.59, 1.73)	1.22 (0.66, 2.26)	0.86 (0.50, 1.49)	0.76 (0.44, 1.30)	1.15 (0.67, 1.97)	0.91 (0.46, 1.82)	1.14 (0.53, 2.45)	1.35 (0.79, 2.29)	0.54 (0.31, 0.93)
Live with spouse (vs. without spouse)	1.05 (0.61, 1.80)	0.77 (0.34, 1.70)	1.51 (0.83, 2.74)	1.03 (0.53, 1.98)	1.16 (0.65, 2.09)	1.07 (0.60, 1.92)	1.01 (0.56, 1.83)	0.61 (0.31, 1.19)	0.44 (0.21, 0.91)	1.02 (0.55, 1.86)	0.90 (0.50, 1.63)
Educational achievement											
≤Middle school	1.00	1.00	1.00	1.00	1.00	1.00	1.00	1.00	1.00	1.00	1.00
High school	1.08 (0.59, 1.99)	0.91 (0.34, 2.42)	1.07 (0.58, 1.99)	1.28 (0.63, 2.59)	1.99 (1.07, 3.73)	1.55 (0.84, 2.86)	1.55 (0.83, 2.88)	1.57 (0.68, 3.64)	0.34 (0.16, 0.73)	1.25 (0.65, 2.39)	1.92 (1.03, 3.56)
≥College	1.01 (0.53, 1.94)	1.33 (0.48, 3.74)	2.49 (1.28, 4.84)	2.15 (1.02, 4.53)	2.64 (1.35, 5.18)	2.16 (1.11, 4.20)	1.07 (0.58, 1.99)	1.56 (0.64, 3.79)	0.30 (0.13, 0.70)	1.29 (0.64, 2.62)	1.90 (0.97, 3.71)
Monthly Household Income											
KRW < 2 million	1.00	1.00	1.00	1.00	1.00	1.00	1.00	1.00	1.00	1.00	1.00
KRW 2–3 million	1.34 (0.74, 2.44)	1.56 (0.59, 4.12)	1.79 (0.95, 3.36)	1.70 (0.85, 3.38)	2.17 (1.16, 4.04)	2.18 (1.17, 4.07)	1.55 (0.83, 2.88)	1.71 (0.79, 3.70)	1.44 (0.61, 3.42)	2.07 (1.09, 3.96)	1.39 (0.74, 2.62)
KRW ≥ 4 million	1.35 (0.75, 2.45)	0.89 (0.33, 2.42)	1.76 (0.95, 3.29)	1.12 (0.57, 2.23)	1.20 (0.65, 2.20)	1.11 (0.54, 2.29)	1.07 (0.58, 1.99)	1.20 (0.55, 2.61)	1.79 (0.76, 4.22)	2.03 (1.08, 3.81)	1.28 (0.68, 2.40)
Smoking status											
Never smoked	1.00	1.00	1.00	1.00	1.00	1.00	1.00	1.00	1.00	1.00	1.00
Ex-smoker	1.28 (0.78, 2.11)	0.94 (0.40, 2.20)	1.05 (0.62, 1.80)	1.79 (0.99, 3.25)	1.49 (0.87, 2.55)	1.28 (0.75, 2.19)	1.33 (0.78, 2.27)	0.94 (0.47, 1.87)	1.05 (0.49, 2.25)	1.19 (0.70, 2.02)	1.26 (0.73, 2.18)
Current smoker	0.88 (0.37, 2.06)	1.02 (0.20, 5.22)	0.83 (0.33, 2.12)	1.24 (0.45, 3.41)	0.79 (0.14, 4.41)	0.58 (0.23, 1.47)	1.05 (0.42, 2.61)	1.15 (0.36, 3.65)	0.68 (0.17, 2.75)	0.57 (0.24, 1.38)	0.73 (0.28, 1.90)
Alcohol consumption											
Not currently drinking	1.00	1.00	1.00	1.00	1.00	1.00	1.00	1.00	1.00	1.00	1.00
Currently drinking	1.13 (0.75, 1.70)	0.28 (0.12, 0.67)	1.14 (0.74, 1.75)	1.00 (0.63, 1.60)	0.96 (0.62, 1.48)	0.94 (0.61, 1.45)	0.71 (0.46, 1.10)	0.61 (0.34, 1.09)	1.69 (0.92, 3.11)	1.11 (0.71, 1.73)	1.54 (0.98, 2.42)
Clinical characteristics											
Time elapsed after diagnosis											
<5 years	1.00	1.00	1.00	1.00	1.00	1.00	1.00	1.00	1.00	1.00	1.00
5–9 years	1.24 (0.39, 3.98)	0.52 (0.10, 2.85)	0.93 (0.26, 3.42)	0.26 (0.07, 0.95)	2.06 (0.50, 8.56)	1.10 (0.30, 4.08)	0.87 (0.25, 3.07)	0.42 (0.11, 1.60)	0.30 (0.08, 1.14)	1.41 (0.38, 5.25)	2.30 (0.57, 9.29)
≥10 years	1.16 (0.37, 3.62)	0.45 (0.09, 2.34)	0.82 (0.23, 2.94)	0.24 (0.07, 0.84)	1.99 (0.49, 8.09)	0.85 (0.23, 3.07)	0.74 (0.22, 2.56)	0.32 (0.09, 1.19)	0.20 (0.05, 0.76)	0.59 (0.17, 2.09)	2.34 (0.59, 9.25)
Continuous increase by 1 year	1.01 (0.96, 1.08)	0.93 (0.83, 1.04)	0.98 (0.92, 1.05)	0.95 (0.89, 1.02)	1.03 (0.96, 1.10)	0.96 (0.90, 1.02)	1.03 (0.96, 1.10)	0.96 (0.88, 1.04)	0.85 (0.75, 0.95)	0.94 (0.88, 1.00)	0.99 (0.92, 1.05)
Cancer stage											
0–1	1.00	1.00	1.00	1.00	1.00	1.00	1.00	1.00	1.00	1.00	1.00
2	1.23 (0.64, 2.34)	0.49 (0.16, 1.45)	1.60 (0.81, 3.16)	0.65 (0.30, 1.43)	1.25 (0.62, 2.55)	1.26 (0.63, 2.53)	1.29 (0.65, 2.58)	1.23 (0.50, 3.04)	2.80 (1.05, 7.47)	1.64 (0.82, 3.27)	1.15 (0.56, 2.35)
3–4	0.67 (0.32, 1.38)	0.51 (0.16, 1.69)	0.86 (0.39, 1.86)	0.78 (0.33, 1.83)	0.75 (0.33, 1.69)	0.96 (0.43, 2.14)	1.56 (0.71, 3.42)	1.13 (0.42, 3.08)	2.92 (0.95, 8.99)	2.07 (0.91, 4.70)	1.01 (0.44, 2.33)
Surgery type *											
Total gastrectomy	1.00	1.00	1.00	1.00	1.00	1.00	1.00	1.00	1.00	1.00	1.00
Biloth-1 subtotal gastrectomy	0.87 (0.55, 1.36)	0.76 (0.38, 1.55)	1.17 (0.72, 1.88)	0.67 (0.40, 1.10)	1.69 (1.04, 2.74)	1.17 (0.73, 1.89)	1.46 (0.91, 2.36)	0.90 (0.50, 1.62)	0.71 (0.37, 1.36)	1.02 (0.63, 1.65)	0.97 (0.59, 1.58)
Biloth-2 subtotal gastrectomy	0.87 (0.47, 1.62)	0.80 (0.29, 2.20)	0.73 (0.38, 1.42)	0.84 (0.42, 1.70)	1.55 (0.80, 3.01)	1.26 (0.65, 2.43)	1.34 (0.69, 2.58)	0.69 (0.28, 1.68)	0.34 (0.11, 1.02)	1.37 (0.68, 2.73)	0.76 (0.39, 1.48)
Pylorus-preserving surgery	0.68 (0.35, 1.32)	0.85 (0.28, 2.56)	0.64 (0.31, 1.33)	0.75 (0.34, 1.63)	1.23 (0.59, 2.56)	1.01 (0.49, 2.08)	0.98 (0.48, 2.02)	0.83 (0.34, 2.05)	1.06 (0.41, 2.73)	1.13 (0.54, 2.37)	1.11 (0.53, 2.31)
Chemotherapy recipient (vs. non-chemotherapy recipient)	1.17 (0.62, 2.19)	3.06 (1.13, 8.29)	0.89 (0.45, 1.73)	1.38 (0.66, 2.91)	1.45 (0.72, 2.93)	1.49 (0.74, 2.99)	0.87 (0.44, 1.72)	0.65 (0.27, 1.57)	0.44 (0.16, 1.21)	0.63 (0.32, 1.22)	0.88 (0.43, 1.77)
Radiotherapy recipient (vs. non-radiotherapy recipient)	1.03 (0.55, 1.94)	0.27 (0.09, 0.80)	0.88 (0.45, 1.70)	0.59 (0.28, 1.23)	0.68 (0.35, 1.33)	0.53 (0.27, 1.05)	0.59 (0.30, 1.15)	0.79 (0.32, 1.92)	0.86 (0.33, 2.23)	1.51 (0.74, 3.12)	1.37 (0.68, 2.77)
Preoperative body mass index											
<23 kg/m^2^	1.00	1.00	1.00	1.00	1.00	1.00	1.00	1.00	1.00	1.00	1.00
23–24.9 kg/m^2^	1.78 (1.13, 2.80)	0.75 (0.34, 1.63)	1.05 (0.64, 1.72)	0.91 (0.53, 1.57)	1.43 (0.87, 2.36)	1.16 (0.71, 1.90)	0.70 (0.43, 1.15)	0.69 (0.37, 1.29)	0.94 (0.45, 1.94)	0.86 (0.53, 1.40)	0.73 (0.44, 1.20)
≥25 kg/m^2^	2.54 (1.66, 3.89)	0.71 (0.34, 1.47)	1.69 (1.08, 2.65)	1.14 (0.70, 1.87)	1.35 (0.85, 2.13)	1.18 (0.75, 1.86)	0.76 (0.49, 1.19)	0.61 (0.34, 1.09)	1.72 (0.94, 3.17)	1.07 (0.68, 1.69)	1.06 (0.66, 1.70)
Continuous increase by 1 kg/m^2^	1.08 (1.01, 1.14)	0.94 (0.85, 1.04)	1.04 (0.98, 1.11)	1.05 (0.97, 1.12)	1.03 (0.97, 1.10)	1.04 (0.98, 1.11)	0.96 (0.90, 1.03)	0.97 (0.90, 1.05)	1.15 (1.05, 1.26)	1.00 (0.94, 1.07)	1.03 (0.96, 1.10)
Psychological characteristics											
High fear of cancer recurrence, FCRI ≥ 13 (vs. FCRI < 13)	1.07 (0.71, 1.61)	1.05 (0.55, 2.01)	1.16 (0.76, 1.76)	1.58 (1.01, 2.46)	1.37 (0.90, 2.09)	1.76 (1.15, 2.70)	1.49 (0.98, 2.27)	1.16 (0.96, 2.71)	0.65 (0.35, 1.22)	1.30 (0.82, 2.04)	1.66 (1.06, 2.58)
Depression, HADS-D ≥ 8 (vs. HADS-D < 8)	1.55 (1.07, 2.24)	0.96 (0.53, 1.76)	1.74 (1.19, 2.56)	0.92 (0.60, 1.41)	1.17 (0.79, 1.72)	1.24 (0.84, 1.82)	0.95 (0.65, 1.40)	0.84 (0.51, 1.38)	1.31 (0.76, 2.26)	1.08 (0.72, 1.62)	1.70 (1.14, 2.54)
Anxiety, HADS-A ≥ 8 (vs. HADS-A < 8)	1.03 (0.60, 1.76)	1.12 (0.50, 2.50)	1.11 (0.64, 1.95)	1.71 (0.98, 3.01)	1.43 (0.81, 2.53)	0.94 (0.53, 1.65)	0.97 (0.56, 1.70)	0.87 (0.44, 1.72)	2.67 (1.32, 5.42)	1.36 (0.72, 2.54)	1.01 (0.56, 1.85)

FCRI, Fear of Cancer Recurrence Inventory; HADS-D, Hospital Anxiety and Depression Scale—Depression; HADS-A, Hospital Anxiety and Depression Scale—Anxiety. * Not specifically stated subtotal: gastrectomy (n = 4), wedge resection (n = 6), and endoscopic submucosal dissection (n = 3) were not included in this analysis due to the small number. Data are presented as the odds ratio and 95% confidence interval, estimated by multiple logistic regression analysis after adjusting for the age at the time of cancer diagnosis, sex, whether living with spouse, level of educational achievement, income, smoking status, alcohol consumption, time elapsed after diagnosis, cancer stage, surgery type, chemotherapy, radiotherapy, pre-op body mass index, Fear of Cancer Recurrence Inventory, Hospital Anxiety and Depression Scale—Depression, and Hospital Anxiety and Depression Scale—Anxiety. The bold values denote statistical significant at the *p* < 0.05 level.

## Data Availability

Data is unavailable due to privacy or ethical restrictions.

## Supplementary Materials

The following supporting information can be downloaded at: https://www.mdpi.com/article/10.3390/nu15143268/s1, Table S1: Questionnaire to measure dietary pattern change of cancer survivors after cancer treatment; Table S2: Dietary change after cancer diagnosis according to the subtype of surgical treatment: 608 Korean gastric cancer survivors; Table S3: Dietary habit change after cancer diagnosis stratified by sex and age at the cancer diagnosis; Table S4: Dietary habit change after cancer diagnosis stratified by sex and lapse after cancer diagnosis; Table S5: Summary table: Identified characteristics of stomach cancer survivors associated with healthier or unhealthier directional change of food intake; Figure S1: Distribution of healthier dietary change score according to the age at the stomach cancer diagnosis.

## References

[1] Sung H., Ferlay J., Siegel R.L., Laversanne M., Soerjomataram I., Jemal A., Bray F. (2021). Global Cancer Statistics 2020: GLOBOCAN Estimates of Incidence and Mortality Worldwide for 36 Cancers in 185 Countries. CA Cancer J. Clin..

[2] Jung K.W., Won Y.J., Hong S., Kong H.J., Im J.S., Seo H.G. (2021). Prediction of Cancer Incidence and Mortality in Korea, 2021. Cancer Res. Treat..

[3] Kang M.J., Won Y.J., Lee J.J., Jung K.W., Kim H.J., Kong H.J., Im J.S., Seo H.G. (2022). Cancer Statistics in Korea: Incidence, Mortality, Survival, and Prevalence in 2019. Cancer Res. Treat..

[4] Carrillo G.M., Santamaría N.P. (2019). Life after a gastrectomy: Experience of patients with gastric cancer. Enferm. Clin. (Engl. Ed.).

[5] Rha S.Y., Lee H.J., Lee J. (2020). Unmet needs in the physical and daily living domain mediates the influence of symptom experience on the quality of life of gastric cancer patients. Support. Care Cancer.

[6] Jeong S.M., Shin D.W., Lee J.E., Jin S.M., Kim S. (2019). Increased Risk of Osteoporosis in Gastric Cancer Survivors Compared to General Population Control: A Study with Representative Korean Population. Cancer Res. Treat..

[7] Seo G.H., Kang H.Y., Choe E.K. (2018). Osteoporosis and fracture after gastrectomy for stomach cancer: A nationwide claims study. Medicine.

[8] Jones L.W., Demark-Wahnefried W. (2006). Diet, exercise, and complementary therapies after primary treatment for cancer. Lancet Oncol..

[9] Cencioni C., Trestini I., Piro G., Bria E., Tortora G., Carbone C., Spallotta F. (2022). Gastrointestinal Cancer Patient Nutritional Management: From Specific Needs to Novel Epigenetic Dietary Approaches. Nutrients.

[10] Pekmezi D.W., Demark-Wahnefried W. (2011). Updated evidence in support of diet and exercise interventions in cancer survivors. Acta Oncol..

[11] Rock C.L., Doyle C., Demark-Wahnefried W., Meyerhardt J., Courneya K.S., Schwartz A.L., Bandera E.V., Hamilton K.K., Grant B., McCullough M. (2012). Nutrition and physical activity guidelines for cancer survivors. CA Cancer J. Clin..

[12] NCCN (2021). NCCN Guidelines for Patients, Stomach Cancer.

[13] Moazzen S., Cortés-Ibañez F.O., van Leeuwen B.L., Alizadeh B.Z., de Bock G.H. (2020). Assessment of Diet Quality and Adherence to Dietary Guidelines in Gastrointestinal Cancer Survivors: A Cross-Sectional Study. Nutrients.

[14] Mosinski J.D., Kirwan J.P. (2016). Longer-Term Physiological and Metabolic Effects of Gastric Bypass Surgery. Curr. Diab. Rep..

[15] Arakawa R., Febres G., Cheng B., Krikhely A., Bessler M., Korner J. (2020). Prospective study of gut hormone and metabolic changes after laparoscopic sleeve gastrectomy and Roux-en-Y gastric bypass. PLoS ONE.

[16] Tollosa D.N., Tavener M., Hure A., James E.L. (2019). Adherence to multiple health behaviours in cancer survivors: A systematic review and meta-analysis. J. Cancer Surviv..

[17] Ryu S.W., Son Y.G., Lee M.K. (2020). Motivators and barriers to adoption of a healthy diet by survivors of stomach cancer: A cross-sectional study. Eur. J. Oncol. Nurs..

[18] Hoang T., Lee J., Kim J., Park B. (2019). Food Intake Behavior in Cancer Survivors in Comparison With Healthy General Population; From the Health Examination Center-based Cohort. J. Cancer Prev..

[19] Continuous Update Project Expert Report 2018 (2018). Recommendations and Public Health and Policy Implications.

[20] Park S., Ahn J., Kim N.S., Lee B.K. (2017). High carbohydrate diets are positively associated with the risk of metabolic syndrome irrespective to fatty acid composition in women: The KNHANES 2007–2014. Int. J. Food Sci. Nutr..

[21] Song S., Lee J.E., Song W.O., Paik H.Y., Song Y. (2014). Carbohydrate intake and refined-grain consumption are associated with metabolic syndrome in the Korean adult population. J. Acad. Nutr. Diet..

[22] Kang Y., Lee K., Lee J., Kim J. (2020). Grain Subtype and the Combination of Grains Consumed Are Associated with the Risk of Metabolic Syndrome: Analysis of a Community-Based Prospective Cohort. J. Nutr..

[23] Pan W.H., Yeh W.T. (2008). How to define obesity? Evidence-based multiple action points for public awareness, screening, and treatment: An extension of Asian-Pacific recommendations. Asia Pac. J. Clin. Nutr..

[24] Beekman E., Verhagen A. (2018). Clinimetrics: Hospital Anxiety and Depression Scale. J. Physiother..

[25] Shin J., Goo A., Ko H., Kim J.H., Lim S.U., Lee H.K., Simard S., Song Y.M. (2017). Validation Study for the Korean Version of Fear of Cancer Recurrence Inventory. J. Korean Med. Sci..

[26] Simard S., Savard J. (2015). Screening and comorbidity of clinical levels of fear of cancer recurrence. J. Cancer Surviv..

[27] Park B., Lee J., Kim J. (2018). Imbalanced Nutrient Intake in Cancer Survivors from the Examination from the Nationwide Health Examination Center-Based Cohort. Nutrients.

[28] Tan S.Y., Wong H.Y., Vardy J.L. (2021). Do cancer survivors change their diet after cancer diagnosis?. Support. Care Cancer.

[29] World Cancer Research Fund/American Institute for Cancer Research (2018). Continuous Updated Project Diet, Nutrition Physical Activity and Stomach Cancer.

[30] Fang X., Wei J., He X., An P., Wang H., Jiang L., Shao D., Liang H., Li Y., Wang F. (2015). Landscape of dietary factors associated with risk of gastric cancer: A systematic review and dose-response meta-analysis of prospective cohort studies. Eur. J. Cancer.

[31] Gaddy J.A., Radin J.N., Loh J.T., Zhang F., Washington M.K., Peek R.M., Algood H.M., Cover T.L. (2013). High dietary salt intake exacerbates Helicobacter pylori-induced gastric carcinogenesis. Infect. Immun..

[32] Costa B.V.L., Menezes M.C., Oliveira C.D.L., Mingoti S.A., Jaime P.C., Caiaffa W.T., Lopes A.C.S. (2019). Does access to healthy food vary according to socioeconomic status and to food store type? an ecologic study. BMC Public. Health.

[33] Walker M.S., Vidrine D.J., Gritz E.R., Larsen R.J., Yan Y., Govindan R., Fisher E.B. (2006). Smoking relapse during the first year after treatment for early-stage non-small-cell lung cancer. Cancer Epidemiol. Biomarkers Prev..

[34] World Cancer Research Fund/American Institute for Cancer Research (2007). Food, Nutrition, Physical Activity, and Prevention of Cancer: A Global Perspective.

[35] Doyle C., Kushi L.H., Byers T., Courneya K.S., Demark-Wahnefried W., Grant B., McTiernan A., Rock C.L., Thompson C., Gansler T. (2006). Nutrition and physical activity during and after cancer treatment: An American Cancer Society guide for informed choices. CA Cancer J. Clin..

[36] Iqbal A. (2017). Effect of Food on Causation and Prevention of Gastric Cancer. J. Cancer Prev. Curr. Res..

[37] (2003). Fruits and Vegetables, IARC Handbooks of Cancer Prevention Volume 8.

[38] Wang Q., Chen Y., Wang X., Gong G., Li G., Li C. (2014). Consumption of fruit, but not vegetables, may reduce risk of gastric cancer: Results from a meta-analysis of cohort studies. Eur. J. Cancer.

[39] Xie Y., Huang S., Su Y. (2016). Dietary Flavonols Intake and Risk of Esophageal and Gastric Cancer: A Meta-Analysis of Epidemiological Studies. Nutrients.

[40] Fagundes M.A., Silva A.R.C., Fernandes G.A., Curado M.P. (2022). Dietary Polyphenol Intake and Gastric Cancer: A Systematic Review and Meta-Analysis. Cancers.

[41] You J., Sun Y., Bo Y., Zhu Y., Duan D., Cui H., Lu Q. (2018). The association between dietary isoflavones intake and gastric cancer risk: A meta-analysis of epidemiological studies. BMC Public. Health.

[42] Yang D., Wang X., Yuan W., Chen Z. (2019). Intake of Anthocyanins and Gastric Cancer Risk: A Comprehensive Meta-Analysis on Cohort and Case-Control Studies. J. Nutr. Sci. Vitaminol..

[43] Thorning T.K., Raben A., Tholstrup T., Soedamah-Muthu S.S., Givens I., Astrup A. (2016). Milk and dairy products: Good or bad for human health? An assessment of the totality of scientific evidence. Food Nutr. Res..

[44] Guo Y., Shan Z., Ren H., Chen W. (2015). Dairy consumption and gastric cancer risk: A meta-analysis of epidemiological studies. Nutr. Cancer.

[45] Zheng G., Sundquist K., Sundquist J., Chen T., Försti A., Hemminki A., Hemminki K. (2021). Second Primary Cancers After Gastric Cancer, and Gastric Cancer as Second Primary Cancer. Clin. Epidemiol..

[46] Nicklett E.J., Kadell A.R. (2013). Fruit and vegetable intake among older adults: A scoping review. Maturitas.

[47] Nakazono M., Aoyama T., Komori K., Watanabe H., Kano K., Nagasawa S., Segami K., Tamagawa H., Yukawa N., Rino Y. (2023). The Comparison of the Dietary Intake Loss Between Elderly and Non-Elderly Patients After Gastrectomy for Gastric Cancer. J. Gastrointest. Cancer.

[48] Thorpe M.G., Milte C.M., Crawford D., McNaughton S.A. (2019). Education and lifestyle predict change in dietary patterns and diet quality of adults 55 years and over. Nutr. J..

[49] McMaughan D.J., Oloruntoba O., Smith M.L. (2020). Socioeconomic Status and Access to Healthcare: Interrelated Drivers for Healthy Aging. Front. Public. Health.

[50] Zajacova A., Lawrence E.M. (2018). The Relationship Between Education and Health: Reducing Disparities Through a Contextual Approach. Annu. Rev. Public. Health.

[51] Ruiz-Tovar J., Bozhychko M., Del-Campo J.M., Boix E., Zubiaga L., Muñoz J.L., Llavero C. (2018). Changes in Frequency Intake of Foods in Patients Undergoing Sleeve Gastrectomy and Following a Strict Dietary Control. Obes. Surg..

[52] Brown J., Byers T., Thompson K., Eldridge B., Doyle C., Williams A.M. (2001). Nutrition during and after cancer treatment: A guide for informed choices by cancer survivors. CA Cancer J. Clin..

[53] Séguin Leclair C., Lebel S., Westmaas J.L. (2021). Can Physical Activity and Healthy Diet Help Long-Term Cancer Survivors Manage Their Fear of Recurrence?. Front. Psychol..

[54] Ramp D., Mols F., Ezendam N., Beijer S., Bours M., Winkels R., de Vries J., Seidell J.C., Kampman E., Hoedjes M. (2021). Psychological distress and lower health-related quality of life are associated with need for dietary support among colorectal cancer survivors with overweight or obesity. Support. Care Cancer.

