# Supplementary material for: Factors Associated with Dietary Habit Changes in Korean Stomach Cancer Survivors after Cancer Treatment
Source: Nutrients. 2023 Jul 24;15(14):3268. doi: 10.3390/nu15143268 (PMC10385203; doi:10.3390/nu15143268)
Supplement: Supplementary file 1 [file nutrients-15-03268-s001.zip › nutrients-2476570-supplementary.pdf]

Supplementary Online Content

Supplementary materials

Supplementary Table S1. Questionnaire of dietary habit change after cancer diagnosis

Supplementary Table S2. Dietary change after cancer diagnosis according to the subtype of surgical treatment: 608 Korean gastric cancer survivors

Supplementary Table S3. Dietary habit change after cancer diagnosis stratified by sex and age at the cancer diagnosis

Supplementary Table S4. Dietary habit change after cancer diagnosis stratified by sex and lapse after cancer diagnosis

Supplementary Table S5. Summary table of findings

Supplementary Figure S1. Distribution of healthier dietary change score according to the age at the stomach cancer diagnosis

This supplementary material has been provided by the authors to give readers additional information about their work.

Supplementary Table S1. Questionnaire to measure dietary pattern change of cancer survivors after cancer treatment

1.	Were there any change in the total amount of food intake, after you had finished cancer treatment?					
		①Decreased a lot	②Decreased somewhat	③Did not change	④Increased somewhat	⑤Increased a lot
2.	Were there any change in the frequency of intake of each food item listed below, after you had finished cancer treatment?					
1)	Red meat	①Decreased a lot	②Decreased somewhat	③Did not change	④Increased somewhat	⑤Increased a lot
2)	Poultry	①Decreased a lot	②Decreased somewhat	③Did not change	④Increased somewhat	⑤Increased a lot
3)	Processed meat	①Decreased a lot	②Decreased somewhat	③Did not change	④Increased somewhat	⑤Increased a lot
4)	Fish	①Decreased a lot	②Decreased somewhat	③Did not change	④Increased somewhat	⑤Increased a lot
5)	Vegetable	①Decreased a lot	②Decreased somewhat	③Did not change	④Increased somewhat	⑤Increased a lot
6)	Fruit	①Decreased a lot	②Decreased somewhat	③Did not change	④Increased somewhat	⑤Increased a lot
7)	Legume	①Decreased a lot	②Decreased somewhat	③Did not change	④Increased somewhat	⑤Increased a lot
8)	Dairy product	①Decreased a lot	②Decreased somewhat	③Did not change	④Increased somewhat	⑤Increased a lot
9)	Grains	①Decreased a lot	②Decreased somewhat	③Did not change	④Increased somewhat	⑤Increased a lot
10)	Salt	①Decreased a lot	②Decreased somewhat	③Did not change	④Increased somewhat	⑤Increased a lot
11)	Burnt food	①Decreased a lot	②Decreased somewhat	③Did not change	④Increased somewhat	⑤Increased a lot

Supplementary Table S2. Dietary change after cancer diagnosis according to the subtype of surgical treatment: 608 Korean gastric cancer survivors

	Total gastrectomy (n=142)		Biloth-1 subtotal gastrectomy (n=328)		Biloth-2 subtotal gastrectomy (n=75)		Pylorus preserving gastrectomy (n=63)		
Dietary component	Decreased	Sustained/ Increased	Decreased	Sustained/ Increased	Decreased	Sustained/ Increased	Decreased	Sustained/ Increased	*p* for difference
Total food	105 (73.9)	37 (26.1)	212 (64.6)	116 (35.4)	53 (70.7)	22 (29.3)	50 (79.4)	13 (20.6)	0.050
Red meat	81 (57.0)	61 (43.0)	187 (57.0)	141 (43.0)	42 (56.0)	33 (44.0)	32 (50.8)	31 (49.2)	0.831
Poultry	67 (47.2)	75 (52.8)	168 (51.2)	160 (48.8)	35 (46.7)	40 (53.3)	32 (50.8)	31 (49.2)	0.809
Processed meat	64 (45.1)	78 (54.9)	152 (46.3)	176 (53.7)	28 (37.3)	47 (62.7)	21 (33.3)	42 (66.7)	0.170
Fish	27 (19.0)	115 (81.0)	56 (17.1)	272 (82.9)	12 (16.0)	63 (84.0)	17 (27.0)	46 (73.0)	0.284
Vegetable	11 (7.7)	131 (92.3)	29 (8.8)	299 (91.2)	5 (6.7)	70 (93.3)	9 (14.3)	54 (85.7)	0.399
Fruit	13 (9.2)	129 (90.8)	31 (9.5)	297 (90.5)	5 (6.7)	70 (93.3)	6 (9.5)	57 (90.5)	0.896
Legume	12 (8.5)	130 (19.5)	20 (6.1)	308 (93.9)	5 (6.7)	70 (93.3)	7 (11.1)	56 (88.9)	0.495
Dairy product	38 (26.8)	104 (73.2)	86 (26.2)	242 (73.8)	17 (22.7)	58 (77.3)	16 (25.4)	47 (74.6)	0.921
Grains	21 (14.8)	121 (85.2)	42 (12.8)	286 (87.2)	5 (6.7)	70 (93.3)	11 (17.5)	52 (82.5)	0.242
Salt	98 (69.0)	44 (31.0)	225 (68.6)	103 (31.4)	55 (73.3)	20 (26.7)	45 (71.4)	18 (28.6)	0.856
Burnt food	83 (58.5)	59 (41.5)	180 (54.9)	148 (45.1)	37 (49.3)	38 (50.7)	33 (52.4)	30 (47.6)	0.611

Data are presented as number (%). *p* for difference were estimated between subtype of surgical treatment

Supplementary Table S3. Dietary habit change after cancer diagnosis stratified by sex and age at the cancer diagnosis

	Age at diagnosis	Dietary change	Total food	Red meat	Poultry	Processed meat	Fish	Vegetable	Fruit	Legume	Dairy product	Grains	Salt	Burnt food
Total (n=624)	< 45 years (n=138)	Sustained/ Increased	38 (27.5)	69 (50.0)	78 (56.5)	53 (38.4)	115 (83.3)	135 (97.8)	134 (97.1)	134 (97.1)	100 (72.5)	119 (83.2)	35 (25.4)	42 (30.4)
		Decreased	100 (72.5)	69 (50.0)	60 (43.5)	85 (61.6)	23 (16.7)	3 (2.2)	4 (2.9)	4 (2.9)	38 (27.5)	19 (13.8)	103 (74.6)	96 (69.6)
	45-54 years (n=231)	Sustained/ Increased	74 (32.0)	100 (43.3)	120 (51.9)	123 (53.2)	187 (81.0)	211 (91.3)	213 (92.2)	215 (93.1)	165 (71.4)	204 (88.3)	63 (27.3)	88 (38.1)
		Decreased	157(68.0)	131 (56.7)	111 (48.1)	108 (46.8)	44 (19.0)	20 (8.7)	18 (7.8)	16 (6.9)	66 (28.6)	27 (11.7)	168 (72.7)	143 (61.9)
	≥ 55 years (n=255)	Sustained/ Increased	86 (33.7)	108 (42.4)	119 (46.7)	180 (70.6)	208 (81.6)	223 (87.5)	221 (86.7)	231 (90.6)	202 (79.2)	220 (86.3)	94 (36.9)	155 (60.8)
		Decreased	169 (66.3)	147 (57.6)	136 (53.3)	75 (29.4)	47 (18.4)	32 (12.5)	34 (13.3)	24 (9.4)	53 (20.8)	35 (13.7)	161 (63.1)	100 (39.2)
		P for difference	0.450	0.316	0.159	**<0.001**	0.846	**0.002**	**0.002**	0.055	0.109	0.762	**0.022**	**<0.001**
Male (n=362)	< 45 years (n=60)	Sustained/ Increased	12 (20.0)	26 (43.3)	31 (51.7)	25 (41.7)	50 (83.3)	59 (98.3)	58 (96.7)	59 (98.3)	45 (75.0)	51 (85.0)	13 (21.7)	9 (15.0)
		Decreased	48 (80.0)	34 (56.7)	29 (48.3)	35 (58.3)	10 (16.7)	1 (1.7)	2 (3.3)	1 (1.7)	15 (25.0)	9 (15.0)	47 (78.3)	51 (85.0)
	45-54 years (n=143)	Sustained/ Increased	48 (33.6)	54 (37.8)	66 (46.2)	71 (49.7)	116 (81.1)	130 (90.9)	132 (92.3)	135 (94.4)	102 (71.3)	126 (88.1)	41 (28.7)	47 (32.9)
		Decreased	95(66.4)	89 (62.2)	77 (53.8)	72 (50.3)	27 (18.9)	13 (9.1)	11 (7.7)	8 (5.6)	41 (28.7)	17 (11.9)	102 (71.3)	96 (67.1)
	≥ 55 years (n=159)	Sustained/ Increased	49 (30.8)	64 (40.3)	74 (46.5)	106 (66.7)	131 (82.4)	140 (88.1)	139 (87.4)	146 (91.8)	130 (81.8)	138 (86.8)	61 (38.4)	88 (55.3)
		Decreased	110 (69.2)	95 (59.7)	85 (53.5)	53 (33.3)	28 (17.6)	19 (11.9)	20 (12.6)	13 (8.2)	29 (18.2)	21 (13.2)	98 (61.6)	71 (44.7)
		P for difference	0.152	0.750	0.752	**0.001**	0.921	0.062	0.079	0.189	0.098	0.829	**0.036**	**<0.001**
Female (n=262)	< 45 years (n=78)	Sustained/ Increased	26 (33.3)	43 (55.1)	47 (60.3)	28 (35.9)	65 (83.3)	76 (97.4)	76 (97.4)	75 (96.2)	55 (70.5)	68 (87.2)	22 (28.2)	33 (42.3)
		Decreased	52 (66.7)	35 (44.9)	31 (39.7)	50 (64.1)	13 (16.7)	2 (2.6)	2 (2.6)	3 (3.8)	23 (29.5)	10 (12.8)	56 (71.8)	45 (57.7)
	45-54 years (n=88)	Sustained/ Increased	26 (29.5)	46 (52.3)	54 (61.4)	52 (59.1)	71 (80.7)	81 (92.0)	81 (92.0)	80 (90.9)	63 (71.6)	78 (88.6)	22 (25.0)	41 (46.6)
		Decreased	62 (70.5)	42 (47.7)	34 (38.6)	36 (40.9)	17 (19.3)	7 (8.0)	7 (8.0)	8 (9.1)	25 (28.4)	10 (11.4)	66 (75.0)	47 (53.4)
	≥ 55 years (n=96)	Sustained/ Increased	37 (38.5)	44 (45.8)	45 (46.9)	74 (77.1)	77 (80.2)	83 (86.5)	82 (85.4)	85 (88.5)	72 (75.0)	82 (85.4)	33 (34.4)	67 (69.8)
		Decreased	59 (61.5)	52 (54.2)	51 (53.1)	22 (22.9)	19 (19.8)	13 (13.5)	14 (14.6)	11 (11.5)	24 (25.0)	14 (14.6)	63 (65.6)	29 (30.2)
		P for difference	0.432	0.447	0.089	<**0.001**	0.857	**0.034**	**0.019**	0.190	0.782	0.809	0.364	**<0.001**

Data are presented as number (%). Bold values denote statistical significant at the p<0.05 level

Supplementary Table S4. Dietary habit change after cancer diagnosis stratified by sex and lapse after cancer diagnosis

	Lapse after diagnosis	Dietary change	Total food	Red meat	Poultry	Processed meat	Fish	Vegetable	Fruit	Legume	Dairy product	Grains	Salt	Burnt food
Total (n=624)	< 5 years (n=166)	Sustained/ Increased	45 (27.1)	70 (42.2)	79 (47.6)	94 (56.6)	127 (76.5)	142 (85.5)	143 (86.1)	146 (88.0)	124 (74.7)	137 (82.5)	33 (19.9)	76 (45.8)
		Decreased	121 (72.9)	96 (57.8)	87 (52.4)	72 (43.4)	39 (23.5)	24 (14.5)	23 (13.9)	20 (12.0)	42 (25.3)	29 (17.5)	133 (80.1)	90 (54.2)
	5- 9 years (n=395)	Sustained/ Increased	131 (33.2)	178 (45.1)	208 (52.7)	223 (56.5)	329 (83.3)	368 (93.2)	366 (92.7)	373 (94.4)	296 (74.9)	345 (87.3)	133 (33.7)	174 (44.1)
		Decreased	264 (66.8)	217 (54.9)	187 (47.3)	172 (43.5)	66 (16.7)	27 (6.8)	29 (7.3)	22 (5.6)	99 (25.1)	50 (12.7)	262 (66.3)	221 (55.9)
	≥ 10 years (n=63)	Sustained/ Increased	22 (34.9)	29 (46.0)	30 (47.6)	39 (61.9)	54 (85.7)	59 (93.7)	59 (93.7)	61 (96.8)	47 (74.6)	61 (96.8)	26 (41.3)	35 (55.6)
		Decreased	41 (65.1)	34 (54.0)	33 (52.4)	24 (38.1)	9 (14.3)	4 (6.3)	4 (6.3)	2 (3.2)	16 (25.4)	2 (3.2)	37 (58.7)	28 (44.4)
		P for difference	0.315	0.789	0.476	0.714	0.114	**0.011**	**0.036**	**0.011**	0.997	**0.015**	**0.001**	0.235
Male (n=362)	< 5 years (n=101)	Sustained/ Increased	24 (23.8)	39 (38.6)	44 (43.6)	53 (52.5)	79 (78.2)	88 (87.1)	88 (87.1)	92 (91.1)	78 (77.2)	84 (83.2)	18 (17.8)	38 (37.6)
		Decreased	77 (76.2)	62 (61.4)	57 (56.4)	48 (47.5)	22 (21.8)	13 (12.9)	13 (12.9)	9 (8.9)	23 (22.8)	17 (16.8)	83 (82.2)	63 (62.4)
	5- 9 years (n=223)	Sustained/ Increased	70 (31.4)	87 (39.0)	109 (48.9)	126 (56.5)	185 (83.0)	205 (91.9)	205 (91.9)	211 (94.6)	168 (75.3)	194 (87.0)	81 (36.3)	89 (39.9)
		Decreased	153 (68.6)	136 (61.0)	114 (51.1)	97 (43.5)	38 (17.0)	18 (8.1)	18 (8.1)	12 (5.4)	55 (24.7)	29 (13.0)	142 (63.7)	134 (60.1)
	≥ 10 years (n=38)	Sustained/ Increased	15 (39.5)	18 (47.4)	18 (47.4)	23 (60.5)	33 (86.8)	36 (94.7)	36 (94.7)	37 (97.4)	31 (81.6)	37 (97.4)	16 (42.1)	17 (44.7)
		Decreased	23 (60.5)	20 (52.6)	20 (52.6)	15 (39.5)	5 (13.2)	2 (5.3)	2 (5.3)	1 (2.6)	7 (18.4)	1 (2.6)	22 (57.9)	21 (55.3)
		P for difference	0.158	0.599	0.674	0.657	0.422	0.260	0.260	0.301	0.690	0.085	**0.001**	0.746
Female (n=262)	< 5 years (n=65)	Sustained/ Increased	21 (32.3)	31(47.7)	35 (53.8)	41 (63.1)	48 (73.8)	54 (83.1)	55 (84.6)	54 (83.1)	46 (70.8)	53 (81.5)	15 (23.1)	38 (58.5)
		Decreased	44 (67.7)	34 (52.3)	30 (46.2)	24 (36.9)	17 (26.2)	11 (16.09)	10 (15.4)	11 (16.9)	19 (29.2)	12 (18.5)	50 (76.9)	27 (41.5)
	5- 9 years (n=172)	Sustained/ Increased	61 (35.5)	91 (52.9)	99 (57.69)	97 (56.4)	144 (83.7)	163 (94.8)	161 (93.6)	162 (94.2)	128 (74.4)	151 (87.8)	52 (30.2)	85 (49.4)
		Decreased	111 (64.5)	81 (47.1)	73 (42.4)	75 (43.6)	28 (16.3)	9 (5.2)	11 (6.4)	10 (5.8)	44 (25.6)	21 (12.2)	120 (69.8)	87 (50.6)
	≥ 10 years (n=25)	Sustained/ Increased	7 (28.0)	11 (44.0)	12 (48.0)	16 (64.0)	21 (84.0)	23 (92.0)	23 (92.0)	24 (96.0)	16 (64.0)	24 (96.0)	10 (40.0)	18 (72.0)
		Decreased	18 (72.0)	14 (56.0)	13 (52.0)	9 (36.0)	4 (16.0)	2 (8.0)	2 (8.0)	1 (4.0)	9 (36.0)	1 (4.0)	15 (60.0)	7 (28.0)
		P for difference	0.723	0.601	0.628	0.554	0.206	**0.015**	0.092	**0.016**	0.516	0.165	0.264	0.073

Data are presented as number (%). Bold values denote statistical significant at the $p<0.05$ level

Supplementary Table S5. Summary table: Identified characteristics of stomach cancer survivors associated with healthier or unhealthier directional change of food intake

		Direction	Relevant food items
Sociodemographic factor	Older age	Unhealthier	Poultry, processed meat, vegetable, fruit, legume, burnt food
	Female	Unhealthier	Burnt food
	Live with spouse	Unhealthier	Grains
	Higher education achievement	Healthier	Processed meat, vegetable, fruit, burnt food
		Unhealthier	Grains
	Higher household Income	Healthier	Vegetable, fruit, salt
Lapse after cancer diagnosis	Longer	Unhealthier	Fish, grains, salt
Preoperative body mass index	Higher	Healthier	Red meat, processed meat, grains
Psychological factor	Higher fear of cancer recurrence	Healthier	Fish, fruit
	Depression	Healthier	Red meat, processed meat, burnt food
	Anxiety	Healthier	Grains

Supplementary figure 1. Distribution of healthier dietary change score according to the age at the stomach cancer diagnosis

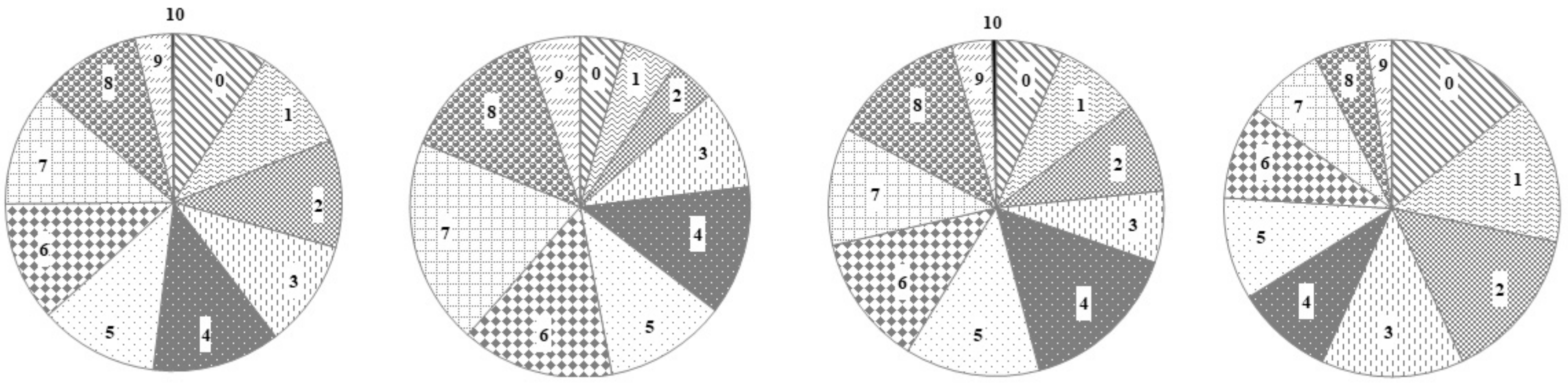

Total subjects | < 45 years old | 45 ~ < 55 years old | ≥ 55 years old

Proportion (%) / Healthier dietary change score*		0	1	2	3	4	5	6	7	8	9	10	11	Mean	*p*-value†
Total Subjects	(n=624)	9.1	9.9	10.3	10.3	12.3	11.4	11.5	11.5	9.9	3.5	0.2	0.0	4.3	
< 45 years old	(n=138)	4.3	5.1	4.3	9.4	12.3	11.6	14.5	19.6	13.8	5.1	0.0	0.0	5.3	<0.001
45 ~ < 55 years old	(n=231)	6.5	8.2	8.7	6.9	15.6	13.0	12.6	11.3	13.0	3.9	0.4	0.0	4.7	
≥ 55 years old	(n=255)	14.1	14.1	14.9	13.7	9.4	9.8	9.0	7.5	5.1	2.4	0.0	0.0	3.4	

*Healthier dietary change score was operationally defined as the number of dietary component in dietary habit changes toward healthier direction

†Assessed by Mantel Henzel chi-square test
